# Violence Exposure Status of Prehospital Care Emergency Medical Services Personnel and the Effects of Violence on the System

**DOI:** 10.34172/aim.34623

**Published:** 2025-09-01

**Authors:** Ali Ekşi, Süreyya Gümüşsoy, Gülseren Keskin, Sezgin Durmuş, Efe Uyanık, Nilüfer Emen, Bektaş Sarı, Sinem Utanır Altay, Yusuf Ali Altuncı, Derya Şaşman Kaylı, Öner Uslu, Günay Serap Tekinsav Sütcü

**Affiliations:** ^1^Atatürk Health Care Vocational School, Ege University, Izmir, Türkiye; ^2^Department of Disaster Medicine, Institute of Health Sciences, Ege University, Izmir, Türkiye; ^3^Ministry of Health General Directorate of Emergency Health Services, Ankara, Türkiye; ^4^Department of Emergency Medicine, Faculty of Medicine, Ege University, Izmir, Türkiye; ^5^Department of Social Work, Faculty of Health Sciences, Celal Bayar University, Manisa, Türkiye; ^6^Faculty of Education, Ege University, Izmir, Türkiye; ^7^Department of Psychology, Faculty of Letters, Ege University, Izmir, Türkiye

**Keywords:** Emergency care, Emergency medical services, Prehospital emergency care, Workplace violence

## Abstract

**Background:**

Personnel working in prehospital emergency medical services (EMS) frequently encounter violence during their duties. This situation negatively affects the safety of healthcare workers and the delivery of services. The aim of this study was to identify the incidence of violence exposure among prehospital care EMS personnel and to evaluate the impact of violence risk on service provision.

**Methods:**

This descriptive, cross-sectional study was conducted among prehospital care EMS personnel in Turkey, with a total of 501 participants. Data were gathered through a structured questionnaire designed to capture instances of violence exposure.

**Results:**

Totally, 40.1% (201) of participants reported experiencing physical violence from at least one patient during their career, while 26.0% (130) reported experiencing physical violence from at least one patient’s relative during their career. Younger participants, those working in urban areas, and those with higher average daily call-out rates experienced higher levels of violence exposure (*P*<.05). Also, 38.5% of the participants reported instances where they could not intervene for the patient due to the risk of violence, and 51.9% reported instances where they did not intervene for the patient until law enforcement arrived due to the risk of violence.

**Conclusion:**

The incidence of violence exposure among prehospital care EMS personnel is notably high. The high rates of violence in urban areas and the time lost in withdrawing from service and waiting for law enforcement intervention indicate that violence is a significant factor affecting service quality.

## Introduction

 According to the World Health Organization (WHO), violence encompasses physical and verbal abuse, homicide, and emotional, sexual, or racial harassment. Violence inflicts harm on human dignity, the right to life, freedom, and security, personal and family life, the right to health, and social well-being, thereby violating and threatening human rights. Workplace violence affects employees across all sectors, but the healthcare sector is particularly vulnerable. A joint report titled “Violence in the Workplace in the Health Sector,” prepared by the WHO, the International Labor Organization, and the International Council of Nurses, indicates that violence in the healthcare sector accounts for approximately one-fourth of all workplace violence incidents.^1^ The risk of healthcare providers experiencing violence is 35 times higher compared to other sectors.^2^ While the rate of encountering physical violence among healthcare workers can reach up to 35%, the rate of experiencing non-physical violence can be as high as 90%.^3^ Due to the relationship between assault rates and patient contact time, one of the most at-risk groups is prehospital care emergency medical services (EMS) personnel. Most EMS workers report verbal, physical, or sexual assaults at least once a year.^4^ In a study conducted in Switzerland, over 80% of EMS workers reported being attacked one to three times a year.^5^

 The violence experienced by healthcare workers increases their occupational anxieties, undermines their sense of security, and adversely affects their desire for work and professional performance, thereby diminishing their work motivation.^6,7^ Furthermore, the escalating incidents of violence in healthcare services, due to their close socio-cultural ties within the societal structure, not only jeopardize the lives of healthcare workers but also negatively impact all aspects of societal life.^3,8^ Violence against EMS personnel is recognized worldwide as one of the most significant challenges facing the service sector. The nature of EMS work, where personnel provide services to patients on-site and may be confined to the limited space of an ambulance cabin with aggressive patients, exposes them to a higher risk of experiencing violence.^9,10^ Currently, there is limited consensus on the identification, assessment, and resolution of the violence encountered by EMS workers. Violence in EMS is a critical issue affecting service quality globally, and further scientific research is needed to develop solution proposals.^11,12^

 The aim of this study was to identify the incidence of violence exposure among EMS personnel and evaluate the impact of violence risk on service provision within the EMS field.

## Materials and Methods

 The data for this descriptive, cross-sectional study were collected between June and August 2023.

###  Research Population and Sample 

 The population of the study consisted of members of the two largest professional associations for EMS workers in Turkey. These are the Paramedics and Prehospital Medicine Association with 1630 members, and the Association of Emergency Medical Technicians and Technicians with 1482 members, totaling 3112 individuals. Rather than sampling from the population, all members were invited to participate in the study. Collaboration was established with the management boards of the professional associations to facilitate the participation of their members in the study. The OpenEpi program was used to calculate the sample size of this study. The sample size was determined as 343 people from the population of 3112 people with a 50% prevalence, 5% margin of error, and 95% confidence interval with the help of the OpenEpi program. In this study, an invitation to participate was sent to the entire target, and 501 participants agreed to participate

###  Data Collection Methods and Instruments

 The data collection method chosen for this study was a questionnaire. The questionnaire included socio-demographic characteristics, experiences of violence exposure during EMS duties, and interventions for aggressive patients. To assess the comprehensibility of the questionnaire items, expert opinions were obtained from five academics specializing in the field of EMS. Additionally, before commencing data collection, a pilot study was conducted with 15 individuals outside the research group to evaluate the clarity of the questionnaire and the duration of administration. Subsequent adjustments were made based on the feedback received, and then the questionnaire was administered.

###  Data Collection Process

 The data collection instruments of the study were sent via email from a pre-determined reliable email address to the active email addresses used by the personnel. The email sent to the personnel included a voluntary consent form explaining the data collection tool and the scope and purpose of the research. Those who agreed to participate clicked on the “I consent to participate in the study” button before filling out the questionnaire, thus providing digital consent. Those who consented to participate in the study completed the data collection instruments online. To prevent multiple responses, measures such as address blocking were implemented. The maximum time allotted for completing the data collection instruments was set at 10‒15 minutes.

###  Statistical Analysis 

 Statistical analysis was conducted using the IBM SPSS Statistics software version 22.0 (SPSS Inc., Chicago, IL). Frequencies and percentages were used for categorical variables, while arithmetic means and standard deviations were used for continuous variables. The significance between independent and dependent variables was determined using the chi-square test. The assumptions of the chi-square test were checked after the analyses, and no cell had an expected value below 1, while 15% of the cells had an expected value below 5, thus fulfilling the assumptions of the test. Findings were evaluated at a 95% confidence interval, and statistical significance was accepted at *P* < 0.05 for all analyses. All *P* values were not reported in pairs according to the significance threshold of 0.05; they were presented with two significant digits (e.g. *P* = 0.03), and values less than 0.001 were indicated as *P* < 0.001. The distribution of variables for descriptive statistics was assessed using the Shapiro–Wilk test. Those showing a normal distribution were reported as mean ± SD, and those not showing a normal distribution were reported as median (IQR). A multivariate logistic regression analysis was performed to control for potential confounding effects. Predefined variables such as age, length of professional experience, station location (urban vs. rural), and daily case count were simultaneously included in the model. Findings are reported as adjusted odds ratios (ORs) and 95% confidence intervals (CIs).

## Ethical Considerations

 The study was conducted in accordance with the principles of the Helsinki Declaration. Prior to the commencement of the study, written approval was obtained from the university’s scientific research ethics committee. All individuals who agreed to participate in the research were explicitly informed that their participation was voluntary and that their personal information would be kept confidential. Informed consent was obtained from all participants before data collection.

## Results

###  Distribution of the Socio-demographic Characteristics of the Participants

 The findings revealed that 46.0% (233) of the participants were in the age group of 20-29, 50.3% (252) were female, 26.6% (133) had 6‒10 years of professional experience, 73.1% (366) resided in urban areas, and 44.3% (222) encountered daily case numbers between 11‒20 (Table 1).

**Table 1:** Distribution of Participants' Sociodemographic Characteristics

**Variable**	**N**	**Median (IQR)**	**%**
Age			
20-29	233	30 (37-26,5)	46.5
30-39	204		40.7
40 +	64		12.8
Gender			
Man	252		50.3
Woman	249		49.7
Years of professional experience			
0-5	122	10 (15-6)	24.4
6-10	133		26.6
11-15	132		26.4
16-20	88		17.6
21 +	26		5.2
Station on duty			
Urban	366		73.1
Rural	135		26.9
Average number of cases per day			
0-5	87		17.4
6-10	164		32.7
11-20	222		44.3
21 +	28		5.6
Total	501		100

###  Participant Exposure to Violence by Patients and their Relatives

 Totally, 40.1% (201) of the participants reported experiencing physical violence from at least one patient during their career, while 26.0% (130) reported experiencing physical violence from at least one patient’s relative during their career. Except for 8.2% of the participants, all others reported experiencing verbal or physical violence from at least one patient, and except for 9.0%, all others reported experiencing verbal or physical violence from at least one patient’s relative. Furthermore, 38.5% (193) of the participants stated encountering situations where they could not intervene for the patient due to the risk of violence and withdrew, while 51.9% (260) reported instances where they did not intervene for the patient until law enforcement arrived due to the risk of violence (Table 2).

**Table 2:** Distribution of participants' exposure to violence from patients and their relatives

**Variable**	**N**	**%**
Have you been subjected to violence by a patient throughout your career?		
Verbal violence	259	51.7
Physical violence	201	40.1
No	41	8.2
Have you been subjected to violence by a patient's relative during your career?		
Verbal violence	326	65.1
Physical violence	130	26.0
No	45	9.0
During your career, have you ever withdrawn from emergency medical intervention due to the risk of violence by patients or their relatives?		
No	48	9.6
Yes, there were cases where we could not intervene for the patient and withdrew due to the risk of violence.	193	38.5
There were cases where we did not intervene for the patient until the police teams arrived.	260	51.9
Total	501	100

 No significant difference was found between the genders regarding their experience of violence (*P* > 0.05).

###  Relationship Between Age and Other Variables

 A statistically significant difference was observed for the ages of the participants regarding their encounters with violence from patients (*P*< 0.05). Specifically, 51.4% of those subjected to verbal violence from patients fell within the age range of 20‒29, whereas 48.7% of those subjected to physical violence from patients were in the age group of 30‒39 (Table 3).

**Table 3:** Distribution of the Relationship Between Age and Other Variables

**Variables**	**Violence**	***n***	**Mean±SD**	**Median (IQR)**	***P***
Have you been subjected to violence by a patient throughout your career?	Verbal violence	259	30.64 ± 6.2	29 (36‒26)	< 0.001
	Physical violence	201	33.64 ± 6	29 (38‒26)	
Have you been subjected to violence by a patient's relative during your career?	Verbal violence	326	31.6 ± 6.1	30 (37‒26)	0.035
	Physical violence	130	33.27 ± 6.2	31.5 (38‒29)	

 Similarly, a statistically significant difference was noted for the ages of the participants regarding their experiences of violence from patients’ relatives (*P* < 0.05). Notably, 49.4% of individuals who faced verbal violence from patients’ relatives belonged to the age group of 20‒29, while 48.5% of those who encountered physical violence from patients’ relatives were in the age group of 30‒39 (Table 3).

###  Relationship Between Years of Work Experience and Other Variables

 A statistically significant difference was found for years of experience of the participants regarding their experiences of violence from patients (*P* < 0.05). Specifically, 26.3% of individuals who faced verbal violence from patients had 11‒15 years of work experience, whereas 30.9% of those who encountered physical violence from patients had 6‒10 years of work experience (Table 4).

**Table 4:** Distribution of the Relationship Between Year of Employment and Other Variables

**Variables**	**Violence**	***n***	**Mean±SD**	**Median (IQR)**	***P***
Have you been subjected to violence by a patient throughout your career?	Verbal violence	259	9.79 **±**6.1	10 (15‒4)	< 0.001
	Physical violence	201	12.59 **±**5.7	11 (16.5‒9)	
Have you been subjected to violence by a patient's relative during your career?	Verbal violence	326	10.25 **±**5.8	10 (15‒5)	< 0.001
	Physical violence	130	12.91 **±**5.9	12 (17‒9.75)	

 Similarly, a statistically significant difference was observed for years of work experience of the participants regarding their experiences of violence from patients’ relatives (*P* < 0.05). It was found that 26.1% of those who experienced verbal violence from patients’ relatives had 6‒10 and 11‒15 years of work experience, while 33.8% of those who experienced physical violence from patients’ relatives had 11‒15 years of work experience (Table 4).

###  Relationship Between the Working Region and Other Variables

 A statistically significant difference was found for the working regions of the participants regarding their experiences of violence from patients (*P* < 0.05). It was revealed that 68.7% of those who experienced verbal violence from patients worked in urban areas, while 80.6% of those who experienced physical violence from patients worked in urban areas (Table 5).

**Table 5:** Distribution of the Relationship Between the Region Studied and Other Variables

		**Working Region**					
		**Urban**		**Rural**			
**Variables**	**Violence**	**N**	**%**	***n***	**%**	**χ**^2^	***P***
Have you been subjected to violence by a patient throughout your career?	Verbal	178	68.7	81	31.3	8.271	0.04
	Physical	162	80,6	39	19,4		
Have you been subjected to violence by a patient's relative during your career?	Verbal violence	234	71.8	92	28.2	3.938	0.047
	Physical violence	105	80.7	25	19.3		
During your career, have you ever withdrawn from emergency medical intervention due to the risk of violence by patients or their relatives?	No	27	56.3	21	43.8	12.738	0.002

 Similarly, a statistically significant difference was observed for the stations where the participants worked regarding their experiences of violence from patients’ relatives (*P* < 0.05). It was found that 71.8% of those who experienced verbal violence from patients’ relatives worked in urban areas, while 80.7% of those who experienced physical violence from patients’ relatives worked in urban areas (Table 5).

 Moreover, a statistically significant difference was noted for the locations of the stations where the participants worked regarding their instances of refraining from providing emergency medical intervention due to the risk of violence from patients or their relatives (*P* < 0.05). It was determined that 80.3% of cases where intervention was not provided due to the risk of violence occurred in urban areas, while 70.8% of cases where intervention was withheld until law enforcement intervened occurred in urban areas (Table 5).

###  Relationship Between the Daily Case Count and Other Variables

 A statistically significant difference was found for the participants’ average daily case counts regarding their experiences of violence from patients (*P* < 0.05). Specifically, it was found that 38.2% of those who experienced verbal violence from patients had a daily average case count of 6‒10, while 56.7% of those who experienced physical violence from patients had a daily average case count of 11‒20 (Table 6).

**Table 6:** Distribution of the Relationship Between the Average Daily Number of Cases and Other Variables

**Variables**		**Number of cases**									
		**0-5**		**6‒10**		**11‒20**		**+21**			
		***n***	**%**	***n***	**%**	***n***	**%**	***n***	**%**	**χ**^2^	***P***
Have you been subjected to violence by a patient throughout your career?	Verbal violence	50	19.3	99	38.2	95	36.7	15	5.8	19.097	< 0.001
	Physical violence	24	11.9	52	25.9	114	56.7	11	5.5		
During your career, have you ever withdrawn from emergency medical intervention due to the risk of violence by patients or their relatives?	No	16	33.3	15	31.3	13	27.1	4	8.3	16.635	0.011
	There were cases where we could not intervene for the patient due to the risk of violence.	30	15.5	58	30.1	90	46.6	15	7.8		
	There were cases where we did not intervene for the patient until the police teams arrived.	41	15.8	91	35	119	45.8	9	3.5		
Have you been subjected to violence by a patient's relative during your career?	Verbal violence	53	16.3	127	39	131	40.2	15	4.6	22.794	< 0.001
	Physical violence	17	13.1	24	18.4	78	60	11	8.5		

 Similarly, a statistically significant difference was found for the participants’ average daily case counts regarding their experiences of violence from patients’ relatives (*P* < 0.05). It was found that 40.2% of those who experienced verbal violence from patients’ relatives had a daily average case count of 11‒20, while 60.0% of those who experienced physical violence from patients’ relatives had a daily average case count of 11‒20 (Table 6).

 Moreover, a statistically significant difference was found for the participants’ average daily case count regarding their instances of refraining from providing emergency medical intervention due to the risk of violence from patients or their relatives (*P* < 0.05). It was observed that 46.6% of cases where intervention was not provided due to the risk of violence occurred in the group with a daily average of 11‒20 cases, while 45.8% of cases where intervention was withheld until law enforcement intervened also occurred in the group with a daily average of 11‒20 cases (Table 6).

###  Multiple Regression Analysis for Factors Influencing Physical Violence

 Multivariate logistic regression analyses examined predictors of patient-perpetrated and family-perpetrated violence while adjusting for age, years of professional experience, station location, and daily case volume (Table 7).

**Table 7:** Multiple Regression Analysis for Factors İnfluencing Physical Violence

**Variable**	**Patient perpetrated violence OR (95% CI)**	***P***	**Patient's relative violence OR (95% CI)**	***P***
Age (per year increase)	0.984 (0.918–1.056)	0.660	0.966 (0.894–1.042)	0.370
Years of professional experience	1.097 (1.020–1.181)	0.013	1.110 (1.023–1.205)	0.012
Urban station (vs. rural)	1.293 (0.769–2.175)	0.332	1.032 (0.568–1.876)	0.917
Daily case volume – 6–11 (vs. 0–5)	1.141 (0.606–2.149)	0.683	0.670 (0.322–1.393)	0.284
Daily case volume – 11–20	2.280 (1.193–4.359)	0.013	2.000 (0.984–4.067)	0.056
Daily case volume – ≥ 21	1.154 (0.422–3.158)	0.780	2.164 (0.759–6.169)	0.149
Constant	0.241	0.093	0.268	0.155

*Models adjusted for age, years of professional experience, station location (rural = reference), and daily case volume (0–5 cases = reference). OR, odds ratio; CI, confidence interval.

 For patient-perpetrated violence, years of professional experience emerged as a significant predictor: each additional year of experience increased the odds of experiencing violence by approximately 10% (OR = 1.097, 95% CI 1.020–1.181, *P* = .013). Daily case volume also showed a dose–response relationship with risk: compared with providers handling 0–5 cases per day, those with 11–20 cases had more than double the odds of patient-perpetrated violence (OR = 2.280, 95% CI 1.193–4.359, *P* = .013), whereas the 6–11 and ≥ 21 case categories did not reach statistical significance (*P* = .683 and *P* = .780, respectively). Neither age (OR = 0.984, 95% CI 0.918–1.056, *P* = .660) nor urban station location (OR = 1.293, 95% CI 0.769–2.175, *P* = .332) were associated with patient-perpetrated violence (Table 7).

 In the model for violence by the patient’s relatives, years of professional experience was again a significant risk factor (OR = 1.110, 95% CI 1.023–1.205, *P* = .012). Providers seeing 11–20 cases per day demonstrated a twofold increase in the odds of violence by patient’s relatives compared with the 0–5 case group, although this finding approached but did not reach conventional significance (OR = 2.000, 95% CI 0.984–4.067, *P* = .056). Age (OR = 0.966, 95% CI 0.894–1.042, *P* = .370), station location (OR = 1.032, 95% CI 0.568–1.876, *P* = .917) or the 6–11 and ≥ 21 case categories (*P* = .284 and p = .149, respectively) did not predict violence by patient’s relatives (Table 7).

 These findings indicate that professional tenure consistently elevates the risk for both types of workplace violence. A high daily case load, particularly in the mid-range (11–20 cases), further exacerbates the likelihood of aggressive incidents from patients and their families (Table 7).

## Discussion

 EMS personnel are highly exposed to occupational violence. In addition to physical injuries resulting from violence experienced by healthcare workers, the anxiety caused by aggressive behaviors can push them to the brink of burnout. Healthcare personnel who struggle to overcome this anxiety may from time-to-time experience decreased functionality, which can negatively impact the quality of patient care.^13^ This study revealed that over 90% of EMS personnel have been subjected to verbal or physical violence from both patients and their relatives. Particularly noteworthy is the fact that two out of every three EMS personnel have experienced verbal violence from patients’ relatives. Verbal violence is often reported more frequently than other forms of violence and serves as a precursor to subsequent physical violence and bullying. The effects of verbal violence should not be underestimated as it can cause more harm than other forms of violence.^14^ In a study conducted by Lafta and Falah, violence was reported to originate mainly from patients’ relatives, followed by the patients themselves.^15^ Another study found that the majority of attacks against healthcare personnel were attributed to patients’ relatives, with 89.7% being verbal and 90.5% being physical assaults.^16^ Consistent with the literature, our study also revealed a high prevalence of violence perpetrated by patients’ relatives. This can be attributed to anxious relatives often preferring to stay in the same area as the patient and being in constant contact with EMS personnel, as evidenced by our findings.

 In the literature, male healthcare workers are reported to experience more physical violence in the workplace compared to females.^17,18^ One study indicated that women tend to perceive aggression more frequently as a destructive behavior compared to men.^19^ This may be due to their fear of violent behaviors and lack of confidence in coping with them. Women tend to perceive themselves as ineffective in dealing with aggression due to social stigmatization and stereotypes. Furthermore, it was found that male nurses are more prone to blaming patients for aggressive behavior.^20^ Female healthcare workers have lower tolerance for aggression.^21^ It has been suggested that female employees facing aggressive behavior from patients may be focused on their own distress, which could hinder their ability to respond to external attacks.^22^ In this study, no difference was found between the genders of the participants regarding their experiences of violence. The differences in EMS work environments, the constant exposure to risky situations, and the high prevalence of violence among EMS personnel may have prevented gender-based differences among EMS workers.

 Our study revealed that the majority of those who experienced physical violence from patients and their relatives were in the 30‒39 age group, while those who experienced verbal violence were predominantly in the younger 20‒29 age group. Particularly noteworthy is the low incidence of violence among EMS workers aged 40 and over and those with more than 21 years of experience. As age increases, the rate of experiencing violence decreases. Similarly, this trend is observed with years of service, where an increase in years of service is associated with a decrease in the rate of experiencing violence. Specifically, the majority of those exposed to verbal and physical violence had 6‒10 years of work experience. Studies in the literature have identified that less experienced, younger healthcare workers are at higher risk of verbal and physical violence.^23^ There are also studies indicating that younger healthcare workers are more vulnerable to workplace violence compared to their older colleagues. A study conducted in South Korea found that workplace violence was most experienced by newly graduated nurses.^24^ A younger age may reflect lack of work experience, which could lead to reduced ability to cope with violence. In the field of EMS, where younger individuals often constitute a significant portion of the workforce, the burden of violence may exacerbate stress levels and diminish their capacity to manage incidents of workplace violence.

 The research revealed that participants stationed in urban centers encountered aggressive behavior from patients and their relatives more frequently and faced a higher incidence of both physical and verbal violence. Mental health challenges are prevalent in both urban and rural settings. Nevertheless, the prevalence of mental disorders may exhibit substantial variation between rural and urban areas, potentially influencing the likelihood of exposure to aggressive conduct from patients. Rates of violence exposure are notably higher among healthcare workers operating in urban settings compared to their counterparts in rural areas. Studies have indicated that instances of severe injuries are more prevalent among aggressors in urban centers.^25^ Conversely, research has suggested that healthcare professionals in rural or small-town settings experience lower levels of physical violence from patients or visitors compared to those in urban locales.^18,26^ The heightened pace of work in urban EMS service provision, coupled with increased time pressures,^3^ alongside the findings of this study, which align with existing literature, underscores their heightened vulnerability to violence. This can adversely impact the motivation of EMS workers and the overall quality of EMS services.

 The significant prevalence of violence exposure from patients and their relatives among EMS workers in our study, coupled with the consequential hesitancy to provide services or intervene until law enforcement arrives, is noteworthy. Workplace violence constitutes a grave and globally concerning phenomenon, with such incidents accounting for 15% of all trauma-related fatalities.^27,28^ Epidemiological research has identified emergency departments and EMS environments as particularly high-risk settings for violence against healthcare professionals.^29,30^ While our study underscores the alarming rates of violence exposure among EMS workers, the delays resulting from service withdrawal due to violence risk and awaiting law enforcement assistance directly impede the efficacy of EMS services, directly impacting the fundamental right to life. While a significant portion of the literature rightfully emphasizes the protection of healthcare personnel.^31,32^ the findings of our study also corroborate the notion that violence constitutes a serious societal issue exacerbating health-related problems.^33^

 Exposure to violence, whether brief or prolonged, can have adverse effects on healthcare professionals, manifesting in both physical and psychological repercussions. Healthcare workers often experience feelings of fear and apprehension when dealing with agitated patients due to the potential risk of violence and harm, either to themselves or others. These emotions can escalate into symptoms of post-traumatic stress disorder with increased violence occurrences or repeated instances of physical and verbal aggression.^34,35^ In our study, participants who reported high levels of violence exposure were predominantly stationed at busy ambulance facilities, suggesting that EMS workers may be particularly vulnerable to traumatic experiences.^36^ Another investigation involving EMS personnel revealed that incidents of violence against healthcare workers could contribute to workplace stress and ultimately lead to burnout.^37^ Notably, frequent encounters with aggressive patients or persistent feelings of fear and anxiety may result in the avoidance of patient or family interactions, thereby significantly compromising the quality of patient care.

## Limitations

 When interpreting the findings of this research, it is crucial to acknowledge its limitations. Firstly, the study focused exclusively on EMS workers, potentially limiting the generalizability of the results to other healthcare professionals. Moreover, an important constraint arises from reliance on a self-report scale for assessing validity and reliability. This method hinges on participants’ subjective accounts, which may not always be entirely accurate or precise. Variations in how individuals interpret and utilize the scales based on their social and cultural contexts can introduce constraints concerning the reliability and validity of the responses. Furthermore, the study’s scope is restricted by its timeframe and cross-sectional design.

## Conclusion

 This study clearly demonstrates the high risk of violence among EMS workers, particularly in urban areas where the incidence of violence is high. The time lost due to withdrawal from service and waiting for law enforcement intervention highlights the significant impact of violence on the quality of EMS services. Combatting violence is important not only to protect EMS workers but also to maintain service quality. Therefore, it is recommended to develop training programs aimed at providing experience for young professionals and to implement measures to protect EMS workers from the stress caused by the risk of violence, especially in urban areas.
